# From vector spaces to DRM lists: False Memory Generator, a software for automated generation of lists of stimuli inducing false memories

**DOI:** 10.3758/s13428-024-02425-0

**Published:** 2024-05-06

**Authors:** Marco A. Petilli, Marco Marelli, Giuliana Mazzoni, Michela Marchetti, Luca Rinaldi, Daniele Gatti

**Affiliations:** 1 Department of Psychology, University of Milano-Bicocca, Milan, Italy; 2 NeuroMI, Milan Center for Neuroscience, Milan, Italy; 3 Department of Health, Dynamic and Clinical Psychology, University of Sapienza, Rome, Italy; 4 Department of Psychology, University of Hull, Hull, UK; 5 Department of Brain and Behavioral Sciences, University of Pavia, Pavia, Italy; 6 Cognitive Psychology Unit, IRCCS Mondino Foundation, Pavia, Italy

**Keywords:** DRM, False memory, Vector space, Distributional semantics

## Abstract

The formation of false memories is one of the most widely studied topics in cognitive psychology. The Deese–Roediger–McDermott (DRM) paradigm is a powerful tool for investigating false memories and revealing the cognitive mechanisms subserving their formation. In this task, participants first memorize a list of words (encoding phase) and next have to indicate whether words presented in a new list were part of the initially memorized one (recognition phase). By employing DRM lists optimized to investigate semantic effects, previous studies highlighted a crucial role of semantic processes in false memory generation, showing that new words semantically related to the studied ones tend to be more erroneously recognized (compared to new words less semantically related). Despite the strengths of the DRM task, this paradigm faces a major limitation in list construction due to its reliance on human-based association norms, posing both practical and theoretical concerns. To address these issues, we developed the False Memory Generator (FMG), an automated and data-driven tool for generating DRM lists, which exploits similarity relationships between items populating a vector space. Here, we present FMG and demonstrate the validity of the lists generated in successfully replicating well-known semantic effects on false memory production. FMG potentially has broad applications by allowing for testing false memory production in domains that go well beyond the current possibilities, as it can be in principle applied to any vector space encoding properties related to word referents (e.g., lexical, orthographic, phonological, sensory, affective, etc.) or other type of stimuli (e.g., images, sounds, etc.).

## Introduction

False memory is a topic of broad interest within cognitive and forensic sciences. The interest is rooted in the fact that human memory is not an accurate recorder, but rather a system that actively forgets and transforms its contents depending on contextual information (Vecchi & Gatti, 2020).

**Fig. 1:**
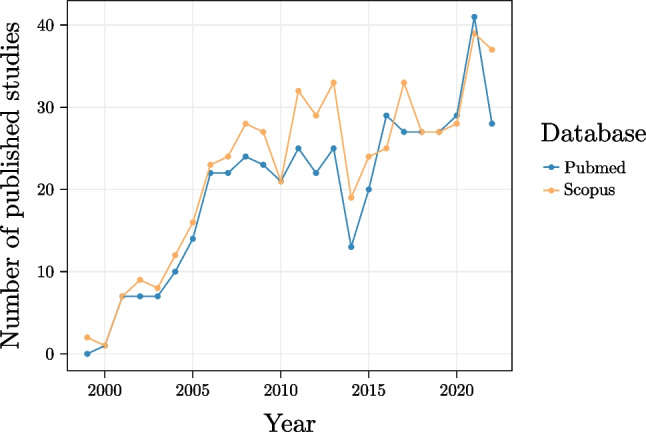
Number of published studies available on PubMed and Scopus using the query “drm AND false memory” between 1999 and 2022

Within the false-memory literature, several tasks have been developed to induce memory distortions and measure humans’ memory performance, with the most widely used task being the Deese–Roediger–McDermott task (DRM; Deese, 1959; Roediger & McDermott, 1995). The success of the DRM can be traced back to the fact that it is an extremely flexible and reliable task and that its results have been reproduced across a high number of different studies (Gallo, 2010). Additionally, the popularity around the DRM task is granted by the fact that this paradigm can be used to test not only the formation of false memory in general, but it can also be taken as a window into broader semantic (but also orthographic, phonological, affective, etc., e.g., Chang & Brainerd, 2021; Sommers & Lewis, 1999) processes. Indeed, a search on PubMed and Scopus shows a steady increase in publications on the topic between 1999 and 2022, now reaching around 500 publications on both scientific search engines (Fig. 1). Note that these numbers refer to the over-conservative query “drm AND false memory” (and that the label DRM started being used after the 1999), with the seminal work of Roediger & McDermott published in 1995 reaching alone over 2800 citations according to Scopus.

The DRM task is divided into three major phases: the encoding phase, the distracting task, and the recognition task. In the encoding phase, participants are first presented with several lists of words that must be memorized (within each list, the words are related to a non-shown target word, named Critical Lure, e.g., word list: *bed*, *rest*, *awake*, *tired*, *dream*, etc. – critical lure: *sleep*). Then, participants are asked to perform a brief distracting task. Finally, in the last phase, participants are shown several words and are asked to indicate which ones were part of the memorized lists (e.g., recognition task). During this latter phase, participants often erroneously recognize as “old” the critical lures (i.e., they recognize them as if they were part of the memorized lists, although these words were never presented during the encoding phase). Interestingly, this effect can be observed across a continuous gradient, with words weakly related to the studied items being generally misrecognized more frequently than unrelated words, but less frequently than critical lures (for a review on the DRM, see Gallo, 2010).

Typically, seminal studies (e.g., Stadler et al., 1999) employed indexes derived from human-based association norms (Nelson et al., 2004), such as the backward associative strength (i.e., the association strength from the words that compose each list to the critical lure; BAS) or the forward associative strength (i.e., the association strength from the critical lure to those that compose each list; FAS), to build the lists of the test. That is, given a word used as critical lure, the (to be studied) words composing its list are arranged in descending order starting from those mostly associated. This procedure presents several constraints. Firstly, from a practical point of view, the association norms should be produced in a specific language of interest to generate (reliable) DRM lists, yet, such norms are currently available only for a small number of languages. In an era where the replicability and cross-cultural generalizability of results are heavily emphasized, this represents a significant constraint. As such, when suitable norms are not available, collecting new association norms should be a necessary preliminary step for constructing DRM lists. This additional process clearly presents limitations in terms of time and effort. Additionally, even when such resources are available, their estimates are time-sensitive, i.e., they depend on the time when ratings were actually collected. Finally, from a theoretical point of view, the association norms are grounded on human-based ratings and several experts argued in favor of the adoption of independent-source measures. That is, in order to improve our knowledge of the cognitive processes underlying an experimental task and avoid circular arguments, it has been argued that future studies should employ predictors built from sources independent from explicit human judgments (Westbury, 2016; for an example of this effect on semantic fluency see: Jones et al., 2015, while for a similar discussion on the DRM task see: Gatti et al., 2022).

Here, to overcome these constraints, we present the False Memory Generator (FMG)—an automated and efficient computerized tool for generating DRM lists starting from a vector space – a mathematical structure where items are represented as numeric vectors in high-dimensional space, enabling quantitative analysis of their relationships. Notably, FMG can create DRM lists based on, in principle, any kind of item – like words in any language or images etc. – as defined by the user in the input vector space. The logic underlying FMG is the same as traditional DRM approaches with a single exception: the relationships between items composing the DRM lists are not (necessarily) constructed on human-based associations but on the similarity relationships between their vector representations (e.g., semantic similarity in a space encoding semantic representations or visual similarity in a space of visual representations). Nevertheless, this single exception is crucial as it also enables using independent sources of information (e.g., corpora of natural language), thus limiting time-consuming and methodological constraints in the construction of the DRM task.

We take as a prominent example distributional semantic models (DSMs). DSMs induce vectors for word meanings through the analysis of word distribution in many linguistic corpora. They are grounded on the distributional hypothesis, according to which words with similar meanings tend to appear in similar contexts (Harris, 1954). Briefly, DSMs represent word meanings as high-dimensional numerical vectors extracted from large amounts of natural language data. Recent DSMs are based on prediction principles (i.e., neural networks that learn to predict a target word based on its lexical contexts) that are consistent with relatively simple, psychologically grounded associative learning mechanisms (Günther et al., 2019; Louwerse, 2018; Rinaldi & Marelli, 2020). Importantly, it is possible to measure the similarity between any word pair included in a vector space by comparing their vector representations. This can be done by calculating a similarity index based on the cosine of the angle formed by the vectors for the two words (the closer the vectors, the higher the cosine; Bullinaria & Levy, 2007; Han et al., 2011). It is crucial to note that DSMs not only represent word meaning based on raw co-occurrence; that is two words will be similar in meaning not because of their mutual co-occurrence score but rather because they have similar global distributional patterns. For example, even if they rarely co-occur, since “surgeon” and “doctor” tend to appear in similar linguistic contexts, and thus the cosine of the angle between their vectors will be higher than the cosine between the vectors of the words “surgeon” and “screwdriver”. In other words, this semantic index is capturing how similar is the way in which two words are used in natural language and, consequently, their meaning. For readers interested in an in-depth discussion of these models, we suggest referring to Günther et al. (2019).

Following this procedure, it is possible to quantify in an objective and data-driven way the similarity relationship between any type of item pair as long as they can be represented within a vector space. Previous studies highlight the validity of applying DSM to DRM tasks (e.g., Chang & Johns, 2023; Gatti et al., 2022; Johns & Jones, 2009; Johns et al., 2012; see also: Osth et al., 2020). Indeed, they have shown that such a similarity metric is a reliable predictor of BAS, where higher similarity between words in a semantic space is associated with increased human-rated associative strength between studied items and the critical lure (Gatti et al., 2022). In addition, they have shown that DSMs can reliably replicate the structure of the DRM task and that the degree of similarity between each new item and the centroid of its list of studied items is a reliable predictor of participants’ performance (Gatti et al., 2022). Specifically, the higher the (semantic) similarity between the new words and the centroid of their lists, the higher the probability of false recognition.

FMG directly exploits such vector space functionality to establish relationships between items, thus enabling to generate DRM lists automatically rather than manually (as would happen with human ratings). Indeed, once the similarities between items in a vector space are established, the tool divides the items into subsets based on their similarities and assigns them to the four possible item conditions for a DRM experiment: (i) Studied Items: items to be presented in the encoding phase of the DRM and, among them, (ii) Target Items: studied items also presented in the recognition phase of the DRM; (iii) Critical Lure(s): new items (i.e., to be presented only in the recognition phase) with higher similarity with the studied items; (iv) Unrelated lures: new items (i.e., to be presented only in the recognition phase) with lower similarity with the studied items.

Here, we present FMG and empirically evaluate its validity and effectiveness via two experiments. First, we exploited FMG to build classical DRM lists (Experiment 1) in which only one critical lure was presented in the recognition phase; second, we considered FMG extended capabilities by testing lists in which the new items were continuously distributed in terms of semantic similarity with the studied items (Experiment 2).

## The FMG software

### Software interface and functionality

The FMG software and a manual detailing the instructions for the FMG are openly available on the OSF platform (https://osf.io/gsrfu). The FMG software is implemented in MATLAB 2022a (The MathWorks, Natick, MA, USA; www. mathworks.com) and compiled into a standalone application for Microsoft Windows with the MATLAB compiler, which makes the MATLAB software and license unnecessary to use FMG.

**Fig. 2:**
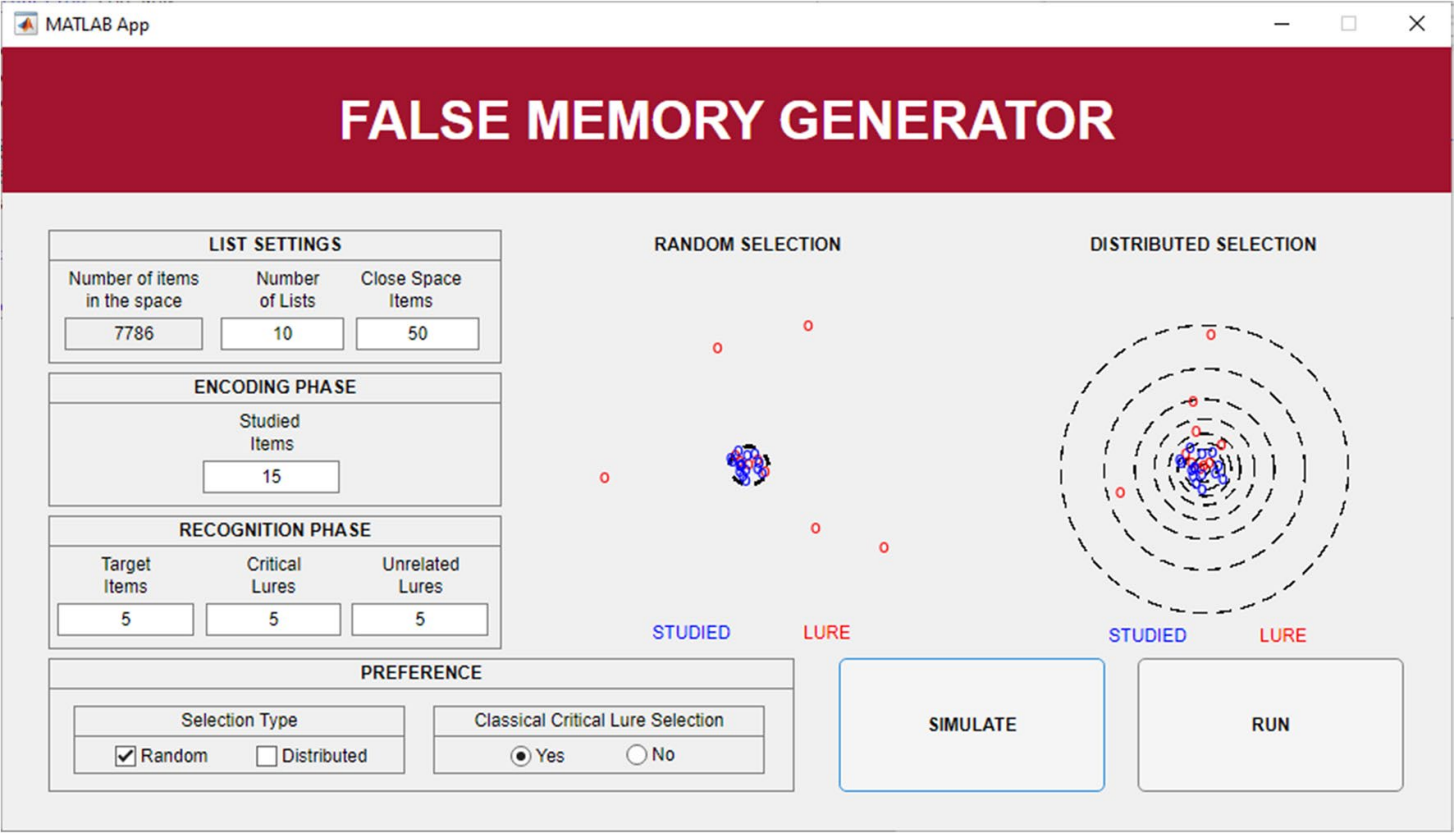
FMG configuration window with parameters to be set and configuration settings for list generation

Once the program is launched, the user has to import a file containing the vector space used to generate lists. Note that the vector space given as input to FMG is determined by the users depending on their specific research focus. For instance, if the user intends to investigate the influence of semantic relationships on the generation of false memory, with a focus on abstract words, the ideal input would be a vector space consisting only of abstract words. The input needs to be a text file, with each line containing a word followed by its vector, each value is space-separated (analogous to the word2vec (Mikolov, Chen, et al., 2013a) or GloVe (Pennington et al., 2014) text embedding format). The user can then specify the criteria for creating the lists by accessing a configuration window (Fig. 2). Here, for validation purposes, we used semantic vectors for words as extracted from DSMs, but FMG accepts any kind of vector space (e.g., encoding orthographic, phonological, visual, and other properties) for any type of items (words, sounds, images, etc.) to be used in a DRM task. For further details on this aspect, please refer to the Discussion section.

The parameters to be set are:

Main list settings:

- Number of Lists (N_L_): this determines the number of lists to be created. Each list will be unique, and items will not appear in multiple lists.
- Close Space Items (N_CS_): This specifies the number of items used to form the *close space* from which *Studied Items* and *Critical Lures* are selected. Note that the value set here must be greater than or equal to the sum of *Studied Items* and *Critical Lures*.

Encoding Phase settings:

- Studied Items (N_S_): This specifies the number of items selected for the encoding phase of each list.

Recognition Phase settings:

- Target Items (N_T_): This specifies the number of *Studied Items* also presented in the recognition phase. This value cannot exceed the number chosen for the *Studied Items* in the encoding phase.
- Critical Lures (N_CL_): This specifies the number of *Critical Lures*: *New Items* positioned close to the centroid of the *Studied Items* (i.e., closer than the farthest *Studied Item*). *Critical Lures* are presented only in the recognition phase. Note that this feature enables to compute more than one *Critical Lure* per list.^1^
- Unrelated Lures (N_UL_): This specifies the number of *Unrelated Lures*: *New Items* positioned far to the centroid of the *Studied Items* (i.e., farther than all the *Studied Items*). *Unrelated Lures* are presented only in the recognition phase.

Then, in the configuration window, the user can customize the following preferences:

- Selection Type: The user can choose whether lists must be generated by applying the random or the distributed methods (see below for a detailed description of the two methods). Only methods that successfully pass a preliminary simulation, testing the feasibility of creating lists with current parameters (for details see the section “List Generation Routine”), can be applied to list generation.
- Classical Critical Lure selection: When selecting “Yes”, FMG will generate lists where the item assumed to be the most critical in the list (i.e., the first neighbor to the centroid of the Studied Items) is selected as a *Critical Lure*. When selecting “No”, this criterion will not be used as a necessary requirement 2.

Then, the user can test through a simulation the compatibility of their setting with the two selection methods implemented in the software (i.e., random and distributed). Each method has specific requirements regarding the size of the whole vector space or the *close space* (for details see the section “Recommendations for configuration” in the FMG tutorial). If one or both methods are incompatible, the software will suggest edits in the configuration window to promote compatibility. After completing these configurations, the user can run the generation of the lists and save the list output in a file with each item and its details listed on a separate line with details about the list to which it belongs, whether it has to be part of the set of items to present in the recognition and/or the encoding phase, and the item type (*Studied Item, Critical Lures,* or *Unrelated Lures*).

#### The list generation routine

**Fig. 3:**
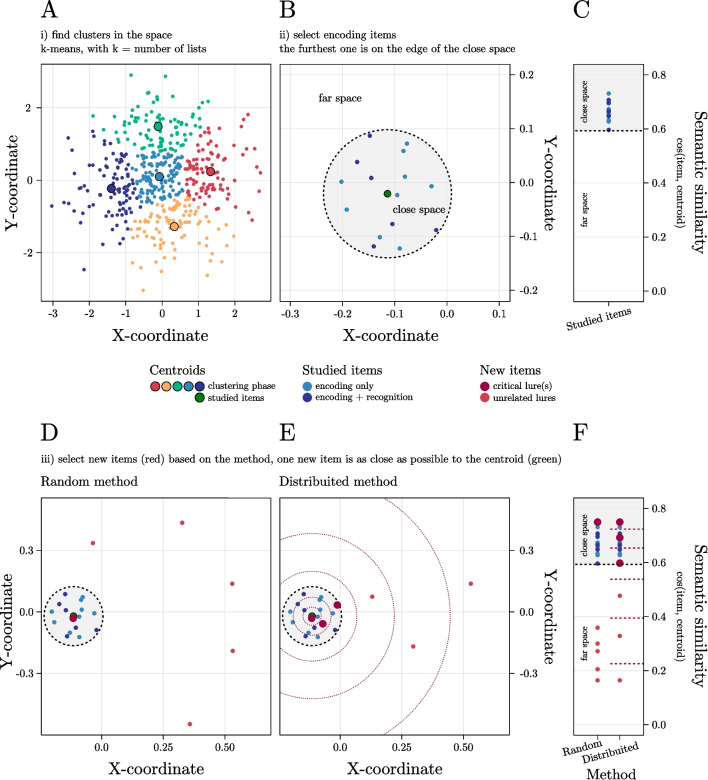
Given a vectorial space, FMG first finds *k* clusters in it (with *k* = number of lists to be created) (A); then, within each cluster, FMG divides the space into close and far (depending on user’s input, see below) and selects the *Studied Items* in the *close space* (B). The distribution of semantic similarity of the *Studied Items* with respect to their centroid is shown in C. Finally, FMG selects the *New Items* among the *close space* (*Critical Lures*) and the *far space* (*Unrelated Lures*) depending on user’s input on numerosity and the chosen method (i.e., Random, D or Distributed, E). The distribution of semantic similarity of all items relative to the centroid of the *Studied Items* is shown in F

The parameters defined in the configuration window determine the routine applied by FMG to the vector space to generate the lists. A graphical representation of this procedure is reported in Fig. 3.

When the list generation routine is run, the software first employs a *k-means* clustering procedure (MacQueen, 1967) to divide the space into clusters according to their similarity. The cluster centroids (i.e., the center of each cluster) are iteratively updated to maximize intra-cluster similarity (grouping similar data points) and minimize inter-cluster similarity (keeping dissimilar data points in separate clusters). As a result, these centroids tend to gravitate towards dense and well-separated regions of the vector space. Note that this procedure provides a stronger guarantee that each of the DRM lists is much more dissimilar to the others. When using the standard approach based on free association norms instead, it is common for some stimulus words to be associated in multiple lists, and these words are not commonly filtered out.

To increase the variability in the items selected across multiple runs, the algorithm employs this procedure to extract a variable number of cluster centroids, ranging from N_L_ to N_L_ + 3. Then the first N_L_ cluster centroids are taken into consideration while the remaining centroids are discarded. By using this procedure, the algorithm extracts a total of N_L_ cluster centroids. Then, each list is generated starting from a distinct cluster centroid.

For each list, first, the vector closest to the cluster centroid is used as center of an initial *close space* (i.e., the portion of the space occupied by the N_CS_ stimuli closest to it). Then, N_S_ stimuli are randomly taken from this portion of the space to form the set of *Studied Items*. The centroid of this set of stimuli is recomputed iteratively until the *Studied Item* farthest to the centroid ends up corresponding to the N_CS_ neighbors, thus matching the user setting. The final *close space* is then defined by the portion of the space occupied by the *Studied Items* so that any stimulus lying in the range space between the encoding set centroid and the farthest *Studied Item* is part of the *close space* (the portion of the space from which *Studied Items* and *Critical Lures* are selected). Otherwise, it is part of the *far space* (the portion of the space from which *Unrelated Lures* are selected).

Note that, in doing these operations, the algorithm aims to fulfil two criteria: (i) maintaining the size of the *close space* as specified in the configuration settings (i.e., therefore, the most peripheral *Studied Items* must correspond to the neighbor of the centroid number N_CS_); (ii) the stimulus closest to the centroid of the *Studied Items* must not include a *Studied Item* itself so that it can be selected as a *New Item* (if desired this option can be disabled by setting “Classical Critical Lure selection” to “No” in the configuration window). However, these conditions may not be satisfied in some cases due to the specific geometrical arrangement of the stimuli in the sub-portion of space at hand. To address this, after 500 iterations in which the criteria are not fully met, FMG restarts the entire selection process, no longer starting from the stimulus vector closest to the centroid, but from the second one (and subsequently by the third closest, and so on, until the conditions are met).

For the recognition phase, N_T_ items are selected randomly from the set of *Studied Items* to form the *Target Items*. The *New Items* (i.e., *Critical* and *Unrelated Lures*) are instead selected depending on the selection methods chosen, as described below.

- Random Method: This method is an adaptation of a procedure used in previous studies (Děchtěrenko et al., 2021; Rodio et al., 2024). According to this procedure, for each list, N_CL_ items are randomly selected among the remaining items in the *close space* (i.e., excluding the *Studied Items* selected) to form the *Critical Lures*. Then, N_UL_ items are randomly selected among the remaining available items (i.e., all items from the space, excluding those lying in all *close spaces* or selected in any form for other lists).
- Distributed Method: This method aims to ensure that the *New Items* are appropriately distributed across the available items. Thus, after selecting the items for the encoding phase, the remaining items in the *close space* (i.e., excluding the *Studied Items*) are sorted based on their distance from the centroid of the encoding set. These items are then divided into N_NL_ portions of increasing amplitude (logarithmically spaced) covering the entire *close space*, so that the dimension of each portion in the sequence is obtained by multiplying the previous one by a fixed ratio. From each of this portion, one item is randomly selected to form a *Critical Lure*. Then the *Unrelated Lures* are randomly selected from portions of the *far space* divided in the same way, thus multiplying the previous portion's dimension by the same fixed ratio. An *Unrelated Lure* is then randomly selected from each of these portions.^3^

**Fig. 4:**
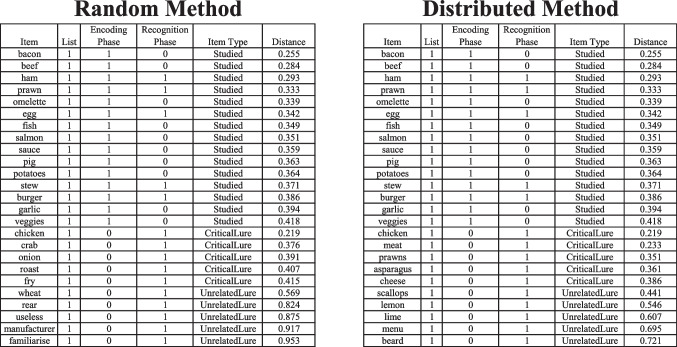
Examples of an output list using the random and the distributed selection method and the configuration parameters as in Fig. 2

Once the list generation process is completed, FMG produces an output file that specifies for each item the list to which it belongs, the phase at which the item is to be presented (i.e., encoding and/or recognition phase), the type of stimulus (i.e., “Studied”, “Critical Lure” or “Unrelated Lure”), and the cosine distance between the item and the centroid of the *Studied Items* (see Fig. 4).

An additional output is automatically generated and reports the entire set of items included in the *close spaces* (as well as those selected in the *far space* as *Unrelated Lures*) of each list sorted according to the distance to the centroid of their respective list. In this way, users can also choose to randomly sample items in the *close space* to be used as *Studied Items/Targets* or *Related Lures* (a practice that is rather common in category length recognition memory designs, e.g., Shiffrin et al., 1995). Indeed, *Studied Items* and *Critical Lures*, being selected from the same region of the space, are both suitable to represent either type of item. However, it is important to be aware that if any changes are made to the set of *Studied Items*, their centroid and distribution in the space will also be altered. As a result, some features of the list generated by FMG – in terms of optimal distance of the selected *Critical Lures* relative to the centroid of the *Studied Items* – may be impacted.

## Experiment 1

In Experiment 1 we used the FMG’s Random Method to generate classical DRM lists and thus to validate its functioning in replicating seminal literature results. That is, each list employed had only one Critical Lure and several Unrelated Lures. Participants were asked to study lists of words and then to perform a recognition task. Consistent with seminal results on the DRM task we expected participants’ false recognition proportions for Critical Lures to be significantly higher than those for Unrelated Lures. Additionally, we also tested whether the model including categorical (Critical Lures vs. Unrelated Lures) vis-à-vis continuous (in terms of distance from the centroid of the list) predictor better indexes participants’ performance.

### Methods

#### Participants

Sixty-four students participated in the study. After removing those not compliant (i.e., those with low accuracy; see Data Analysis section), the final sample included 57 participants (eight males, *M* age = 24.03 years, *SD* = 2.44, age range = 21–33). All participants were native Italian speakers, had normal or corrected to normal vision and were naïve to the purpose of the study. Informed consent was obtained from all participants before the experiment. The protocol was approved by the psychological ethical committee of the University of Pavia (protocol n. 051/23) and participants were treated in accordance with the Declaration of Helsinki.

#### Stimuli

The stimuli were selected starting from the vector representations provided by *fastText* (Bojanowski et al., 2017), a recent DSM. Words vectors for the 7786 Italian dominant lemmas included in the database by Amenta and colleagues (in prep) 4 were retrieved from the Italian pre-trained *fastText* vectors (Grave et al., 2018). The model was trained on Common Crawl (around 630 billion words) and Wikipedia (around 9 billion words) using the Continuous Bag of Words (CBoW) method, an approach originally proposed by (Mikolov, Chen, et al., 2013a), with 300 dimensions and a co-occurrence window of five words.

In this experiment, we used the Random method of FMG. The Number of Lists was set to 12, Close Space Size to 50 items, Studied Items to 15, Target Items to 6, Critical Lures to 1, and Unrelated Lures to 5. In addition, we forced the selection of the closest item to the centroid of the Studied Items as a Critical Lure. The procedure was run until all lists were successfully created, i.e., selecting the closest new item and keeping the close space size as specified in the configuration setting. In total, the recognition phases were composed of 144 words, 72 of which had been presented in the encoding phase (i.e., studied words) and 72 of which had not been presented (i.e., new words).

#### Procedure

During the first part of the task, participants had to memorize a series of words. Participants were shown the 15 words that composed each of the 12 lists in descending cosine similarity with the centroid of the list. The order by which the lists were presented was random, while the order of the words within each list was fixed, as standard in the DRM literature (Roediger & McDermott, 1995). No interruptions were included among the lists, with the words being sequentially presented. Each trial started with a central fixation cross (presented for 500 ms) followed by a word (presented for 1500 ms) and a blank screen (presented for 300 ms), then the script moved automatically to the next fixation cross.

At the end of the encoding phase, participants were required to perform an attentional task (i.e., a modified version of the go-no-go) as a distracting task for 2 min. Then, participants were asked to perform the recognition task. In the recognition task, participants were shown one word at a time and were instructed to judge if the word shown was old or new, that is, if they had been presented in the encoding phase (“old”) or not (“new”). Participants were asked to respond as fast and as accurately as possible by pressing two buttons of a standard keyboard (e.g., A and L) using left and right hands. Each trial started with a central fixation cross (presented for 500 ms) followed by a word (presented until response); after participant’s response, a blank screen (lasting 1000 ms) was presented and then the next trial began.

Participants were tested using PsychoPy (Peirce et al., 2019) through the online platform Pavlovia (https://pavlovia.org/).

#### Data analysis

All the analyses were performed using *R*-Studio (RStudio Team, 2015). Signal-detection measures used to detect non-compliant participants were calculated using the *psycho* R package (Makowski, 2018). Specifically, using “old” responses to studied words and “old” responses to Unrelated Lures we computed participants’ A' values (Donaldson, 1992; Stanislaw & Todorov, 1999); and for a similar method with the DRM, see: Diez et al., 2017; Gatti et al., 2021). A' provides a measure of discriminability (i.e., the ability to discriminate the signal – the words actually studied – from the noise) comprised between 0 and 1, with values around 0.5 indicating chance performance and values around 1 good performance. Participants having A' < 0.7 were removed from the sample.

The main analyses were performed on raw data through a mixed-effects approach, which incorporates both fixed-effects and random-effects (associated to participants and task stimuli) and allows for the specification of predictors at both participants and item level. All the generalized linear mixed models (GLMMs) were run using the *lme4 R* package (Bates et al., 2015). Firstly, we aimed to test whether participants’ false memories were higher for Critical Lures as compared with the Unrelated Lures. We thus estimated a GLMM having participants’ responses (i.e., “new” responses were scored as 0, whereas “old” responses as 1) as dependent variable, the type of item (Studied Word vs. Critical Lure vs. Unrelated Lure) as categorical predictor, and participants and items as random intercepts. Then, we tested whether the generated lists can also be conveniently used in experiments aimed at testing continuous effects. This is especially relevant for the cases in which the Distributed Method cannot be applied due to specific vector space constraints. That is, to this aim, we tested i) whether the semantic similarity between each new word and the centroid of its list explained participants' performance and ii) which model better indexes participants’ behavior. We thus estimated two GLMMs on the subset of new words, one including the similarity with the cosine of the list as continuous predictor and the other including the type of new item (Critical Lure vs. Unrelated Lure) as categorical predictor. Note that this last GLMM was estimated only on new items in order to allow for a comparison with the GLMM including the continuous predictor. Participants and items were set as random intercepts. Model comparison was performed by estimating the Akaike information criterion (which returns an estimation of the quality of the model, the lower the better, AIC; Akaike, 1998) of the two models. A ΔAIC = 2 is generally considered as indicative of evidence in favor of the one with the lower AIC (Hilbe, 2011; as it would indicate that the model with lower AIC is 2.7 times more likely to be a better model in terms of Kullback-Leibler distance from the “real” distribution than the model with higher AIC (Wagenmakers & Farrell, 2004).

#### Results

**Fig. 5:**
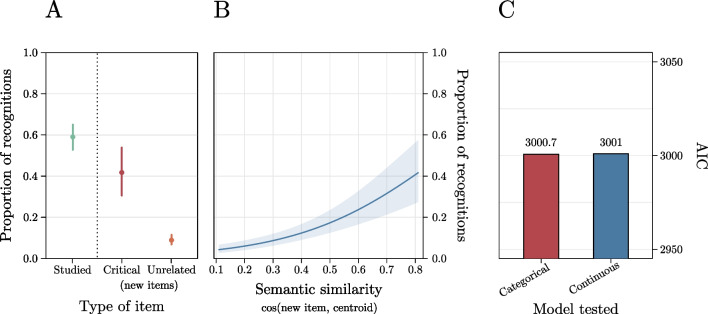
Results of Experiment 1 showing the results of the categorical model including the type of item (**A**), the results of the model including the semantic similarity between each new word and the centroid of its list (**B**), and the comparison of this latter model with the categorical model estimated on the subset of new items (**C**)

Trials in which reaction times were faster than 300 ms or slower than 3000 ms were excluded from the analysis (2.8% of the trials excluded). The first GLMM (*marginal Pseudo-R*^*2*^ = .28; *total Pseudo-R*^*2*^ = .45) showed that the effect of type of item was significant, *χ*^*2*^ = 329, *p* < .001 (Fig. 5A). Consistent with our expectations, post-hoc comparisons showed that participants reported a higher number of false memories for the words that FMG selected as Critical Lures (probability = .41, *SE* = .06) compared with those selected as Unrelated Lures (probability = .08, *SE* = .01), *b* = 1.99, *z* = 7.11, *p* < .001. Participants proportion of recognitions was higher for those selected as Studied Words (probability = .60, *SE* = .03) as compared with both those selected as Critical Lures,* b* = .70, *z* = 2.78, *p* = .01, and as Unrelated Lures,* b* = 2.70, *z* = 18.56, *p* < .001.

Both the GLMMs estimated on the subset of new words showed significant effects. Specifically, the GLMM including the continuous predictor (*marginal Pseudo-R*^*2*^ = .11; *total Pseudo-R*^*2*^ = .42) showed that the effect of semantic similarity between each new word and the centroid of its list was significant, *b* = 3.95, *z* = 6.39, *p* < .001 (Fig. 5B). This indicates that false recognitions increased with increasing semantic similarity between each new word and the centroid of its list, consistent with previous evidence (e.g., Gatti et al., 2022). Similarly, the GLMM including the categorical predictor (*marginal Pseudo-R*^*2*^ = .11; *total Pseudo-R*^*2*^ = .42) showed that the effect of type of new word was significant, with Critical Lures being falsely recognized more often than Unrelated Lures, *b* = 2.15, *z* = 6.47, *p* < .001. These results indicate that using DRM lists as generated through FMG it is possible to reliably test both a (classical) categorical effect of the type of words in the DRM as well as a continuous one. These two models had an AIC of 3001 and 3000.7, respectively, and thus they can be considered as equivalent in explaining participants’ performance (Fig. 5C). Subsequent sensitivity analyses conducted on 1000 simulations revealed a high observed power (95% CI 95–100%) for both models.

## Experiment 2

In Experiment 2, we used FMG’s Distributed Method to generate a new type of DRM lists. That is, each list employed had a continuous distribution of (semantic) similarity among the new items and the studied ones. As for Experiment 1, participants were asked to study lists of words and then to perform a recognition task. We expected to replicate the results of Experiment 1 with participants’ false recognition proportions for close new items to be significantly higher than those for far new items. Additionally, given the structure of the stimuli, we expected the model comprising a continuous predictor to be better in explaining participants’ false recognitions as compared with the model including the categorical predictor (Critical Lures vs. Unrelated Lures).

### Methods

#### Participants

Sixty-six students participated in the study. After removing those non-compliant, the final sample included 58 participants (six males, *M* age = 23.05 years, *SD* = 2.43, age range = 20–34). All participants were native Italian speakers, had normal or corrected to normal vision and were naïve to the purpose of the study. Informed consent was obtained from all participants before the experiment. The protocol was approved by the psychological ethical committee of the University of Pavia and participants were treated in accordance with the Declaration of Helsinki.

#### Stimuli

Stimuli construction was identical to Experiment 1. The only difference was that in Experiment 2 the stimuli were selected using the Distributed method instead of the Random one, and the number of Critical Lures and Unrelated Lures were both set to 3.

#### Procedure

The procedure was identical to Experiment 1.

#### Data analysis

Data analysis was identical to Experiment 1.

### Results

**Fig. 6:**
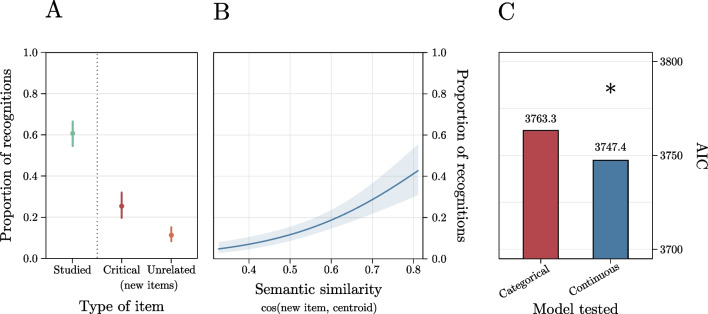
Results of Experiment 2 showing the results of the categorical model including the type of item (**A**), the results of the model including the semantic similarity between each new word and the centroid of its list (**B**), and the comparison of this latter model with the categorical model estimated on the subset of new items (**C**)

Trials with reaction times faster than 300 ms or slower than 3000 ms were excluded from the analysis (1.1% of the trials excluded). The first GLMM (*marginal Pseudo-R*^*2*^ = .21; *total Pseudo-R*^*2*^ = .39) showed that the effect of type of item was significant, *χ*^*2*^ = 211, *p* < .001 (Fig. 6A). Consistent with our expectations, post hoc comparisons showed that participants reported a higher number of false memories for the words that FMG selected as Critical Lures (probability = .25, *SE* = .03) compared with those selected as Unrelated Lures (probability = .11, *SE* = .01), *b* = .97, *z* = 4.71, *p* < .001. Participants proportion of recognitions was higher for those selected as Studied Words (probability = .60, *SE* = .03) as compared with both those selected as Critical Lures,* b* = 1.50, *z* = 8.66, *p* < .001, and as Unrelated Lures,* b* = 2.48, *z* = 13.79, *p* < .001.

Both the GLMMs estimated on the subset of new words showed significant effects. Specifically, the GLMM including the continuous predictor (*marginal Pseudo-R*^*2*^ = .10; *total Pseudo-R*^*2*^ = .35) showed that the effect of semantic similarity between each new word and the centroid of its list was significant, *b* = 5.56, *z* = 5.96,* p* < .001 (Fig. 6B). This indicates that false recognitions increased at increasing semantic similarity between each new word and the centroid of its list, consistent with previous evidence (e.g., Gatti et al., 2022). Similarly, the GLMM including the categorical predictor (*marginal Pseudo-R*^*2*^ = .05; *total Pseudo-R*^*2*^ = .36) showed that the effect of type of new word was significant, with Critical Lures being falsely recognized more often than Unrelated Lures, *b* = 1.01, *z* = 3.73, *p* < .001. These two models had an AIC respectively of 3747 and 3763, and thus since the ΔAIC = 16, the model including the continuous predictor is 2981 times more likely to be a better model than the other^5^ (Fig. 6C). Subsequent sensitivity analyses conducted on 1000 simulations revealed a high observed power (95% CI 95–100%) for both models.

### Discussion

In the present study, we described FMG, the first tool that automatically creates lists for the DRM, a widely used experimental paradigm to induce and investigate false memory production. FMG is an innovative and practical tool for researchers working in the field of (semantic) memory. Unlike traditional methods that rely only on human-produced associations, FMG utilizes data-driven computational approaches (but it is flexible enough to also use human-produced ratings) to establish item relationships by leveraging on the similarity between their representations populating a vector space. By exploiting the capabilities of vector spaces to establish relationships between items, FMG enables the creation of multiple dissimilar DRM lists, reducing the risk of some stimuli being associated with multiple lists. In doing this, FMG eliminates the time-consuming process of constructing DRM lists manually. Indeed, FMG creates DRM lists in an automated manner, bypassing the need for manual and expensive collection of human data. Furthermore, FMG has versatile configuration options that allow researchers to customize list parameters and selection of items, ensuring precise and flexible control over experimental design.

Notably, FMG accepts any input type as long as it is defined in a vector space format. By producing DRM lists optimized on the property encoded in the vector space, FMG allows for testing to what extent that property is critical in inducing false memories. In the present study, we used a DSM to test, across two experiments, the validity of the DRM lists generated through FMG. Specifically, in Experiment 1, employing lists of words reproducing the structure of classical DRM lists, we replicated the seminal evidence provided by Roediger & McDermott (1995), with higher proportion of false memories for Critical Lures as compared with Unrelated Lures. Then, in Experiment 2 we employed a new type of DRM lists characterized by a continuous distribution of the new items in the recognition phase. We thus replicated the semantic similarity effect on false memory production (Gatti et al., 2022), with false recognitions being predicted by the degree of (semantic) similarity between each new item and the ones composing its list. Importantly, the lists built through FMG replicate both effects, thus providing compelling evidence for the validity of FMG as a tool for inducing false memories.

**Fig. 7:**
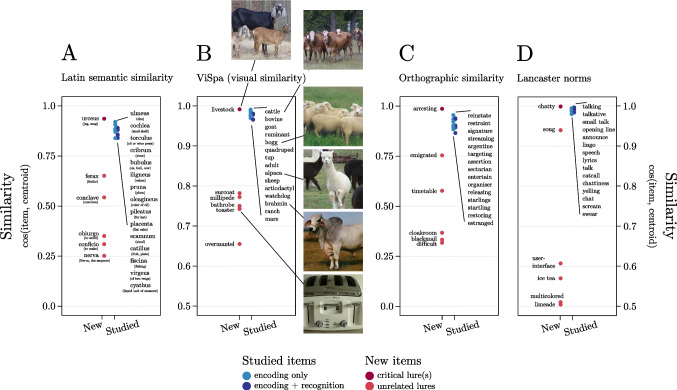
Examples of DRM lists generated using various vector spaces as input for FMG. Each list was created by setting a close space of 50 items, 15 Studied Items, six Targets, one Critical Lure, and five Unrelated Lures, mirroring the configuration settings used in Experiment 1. All items are arranged according to their similarity with the centroid of the studied items. Studied items are represented in *blue* (*light blue* for non-target and dark blue for target). New items are represented in *red* (*dark red* for the Critical Lures and *light red* the Unrelated Lures). **A** This list was generated by providing FMG with lemma embeddings for Latin (Sprugnoli et al., 2019). Embeddings were trained using *fastText* (Bojanowski et al., 2017) with the skip-gram architecture and 100 dimensions. The English translation is provided in parentheses. **B** This list was extracted feeding FMG with the ViSpa, a high dimensional vector space including vision-based representation for word referents (Günther et al., 2022). To provide a visual idea of the selected stimuli, examples of typical images for some of the word categories are provided (from http://vispa.fritzguenther.de). **C** This list was extracted feeding FMG with a vector space of orthographic features of nine-letter words extracted from Subtlex UK (van Heuven et al., 2014). The vector space comprises 26 dimensions, with each dimension representing the presence (indicated by *1*) or absence (indicated by *0*) of a specific letter in the alphabet. **D** This list was extracted from feeding FMG with a vector space having as dimensions sensorimotor strength ratings from the Lancaster Sensorimotor Norms; Lynott et al., 2020

Interestingly, and consistent with the aims of building DRM lists selecting new items with the Distributed Method, the model including the continuous predictor outperformed the model including the categorical one (i.e., the classical view of the DRM task) only in Experiment 2, that is when lists were built using the Distributed Method. This dissociation highlights how the two methods produce lists that differently affect participants’ performance in the DRM task and demonstrates that while the Random Method appears equally suitable for categorical and continuous analyses, the Distributed Method generate lists optimized for analyses that treat item similarity as a continuous predictor. Notably, these two methods have advantages and disadvantages. On the one hand, the Random Method is more suitable for small vector spaces, while the Distributed one generally requires large vector spaces. On the other hand, the Distributed Method fosters a better distribution of items, an aspect that may be critical in the Random Method, especially when dealing with a limited number of items. This method enables a more accurate representation of the impact of similarity on false memory, ensuring that even subtle variations, which might be missed with uncontrolled item distribution, are effectively captured. Thus, when selecting the method, these technical, methodological, and theoretical considerations should be taken into account. FMG offers maximum flexibility in allowing researchers to select and define the most suitable vector space to answer theoretical questions. To date, a wide variety of vector spaces that can potentially be used as input for the FMG to create DRM lists are available. To give an idea, some renowned examples of text embedding models include Word2Vec (Mikolov, Sutskever, et al., 2013b) GloVe (Pennington et al., 2014), fastText (Joulin et al., 2016), ELMo (Peters et al., 2018), and BERT (Devlin et al., 2018) (note that these are just a few examples but there are many others available). The use of FMG, combined with the extensive availability of vector spaces, allows for the testing of various processes that can play a role in false memory formation. Critically, this goes well beyond the current possibilities. Firstly, FMG is a tool that can be applied to multilingual datasets, overcoming the challenges of developing suitable stimulus sets for the DRM across diverse languages. It should be noted that vector spaces already exist in many languages; e.g., the *fastText* platform provides vector representations of words for 157 languages (Bojanowski et al., 2017; https://fasttext.cc/). Any of these vector spaces can be fed to FMG to create DRM lists easily. This makes it easy to investigate the impact of semantic relations on false memories across various languages (see Fig. 7A for an example of DRM list with Latin words).

Secondly, FMG can extend the focus of DRM research beyond the impact of semantic relationships between words. Indeed, it provides a methodological scaffold to explore the role of a wide range of other aspects that could potentially come into play. For example, on a lexical level, it can be hypothesized that other linguistic dimensions, such as phonetics, phonology, morphology, or orthography, may contribute to false memory production (e.g., for evidence on phonological, see Sommers & Lewis, 1999). Vector representations capturing these attributes have already been developed and used in previous research on different topics and can be easily used as input of FMG (e.g., for word segmentation, Ma et al., 2016; recognition, Mortensen et al., 2016; morphological derivation, Marelli & Baroni, 2015, and inflexion, Silfverberg et al., 2018). Feeding FMG with similar vector spaces would allow for the creation of DRM lists optimized to test the role of the property encoded in the vector space at hand, thus offering new possibilities for studying the complex relationship between language features and memory.

Additionally, several other semantic aspects could be examined. For example, within the grounded cognition framework (e.g., Barsalou, 1999, 2008), which emphasizes the importance of perceptual and motor experiences in semantic processing, there is potential to delve deeper into the role of sensorimotor aspects of word referent in memory distortions. To this aim, researchers could utilize vector spaces that encode sensorimotor features as input for FMG (such as Wingfield & Connell, 2022). This would allow for the generation of DRM lists optimized to investigate how sensorimotor representations of concepts influence memory formation and retrieval. The progress in machine learning techniques is further driving the creation of novel vector spaces for this purpose (e.g., LeCun et al., 2015). This is made possible by the ability of these techniques to extract relevant features from sensory stimuli (e.g., images or sounds, e.g., Hershey et al., 2017; Krizhevsky et al., 2012), producing valid and cognitively plausible high-dimensional sensory representations for concepts (Petilli et al., 2021; Zhang et al., 2018). A notable example in this sense is ViSpa (Günther et al., 2022), a recently released high-dimensional vector space that includes vision-based representation for several word referents. These vector representations are derived directly from visual stimuli using a deep convolutional neural network trained to classify images. Such a space could be useful for investigating the role of visually grounded properties of word referents in generating false memories (see Fig. 7B for an example of list generated using the vector space ViSpa as input of FMG).

However, vector spaces used in FMG do not necessarily require advanced and complex machine learning techniques to be created. For example, a vector space capturing orthographic aspects of words can be easily built-up using one-hot encoding, which is vectors of zeros and ones indicating the presence or absence of certain letters in words (see Fig. 7C for an example of list generated feeding FMG with a similar vector space). Or one can opt to directly feed FMG with human judgements as dimensions of a vector space. For instance, within the embodied framework, one can opt to directly feed FMG with perceptual and motor strength ratings to extend the exploration of the role of the sensorimotor experience across various modalities (Lynott et al., 2020; see Fig. 7D for an example of list generated using sensorimotor strength ratings as dimension of the vector space). Indeed, such ratings could be directly employed as dimensions of the vector space, enabling creation of DRM list optimized to capture the sensorimotor distance between concepts (Wingfield & Connell, 2022), i.e., the similarity in the extent to which concept referents can be experienced through various senses (i.e., auditory, gustatory, haptic, interoceptive, olfactory, and visual) or by performing an action with various effectors (the hand/arm, head, foot/leg, mouth, or torso effectors). Note that input spaces of FMG do not necessarily need to include all dimensions of the space under consideration. If the dimensions of the vector space are interpretable, it is possible to select the ones that are particularly relevant to the specific domain the researcher intends to explore. Similarly, one can select subspaces with different combinations of attributes from the "sensorimotor norms" to explore particular aspects, such as the selective role of action experience, to people’s semantic representations.

It is worth highlighting that FMG does not prevent the creation of lists using also classic word association norms. One, for example, can still opt to use a vector space based on the distribution of words in association norms and FMG will produce lists of stimuli optimized to capture the semantic relationships encoded in such space. In principle, one could even use FMG to empirically compare the efficacy of DRM lists created from human intuitions (e.g., word association norms) with those from independent sources (e.g., word distribution in language) in inducing false memories. From a theoretical perspective, the literature suggests that the possibility of using an independent source is highly desirable for psychological studies, as it allows for bypassing the loophole of predicting behavioral data (e.g., false memory) from other behavioral data (e.g., word association intuitions) – which would leave us at the same level of description without actually addressing the cognitive phenomenon of interest (for a more comprehensive explanation of this argument, please refer to the works by Jones et al., 2015; Westbury, 2016; Petilli et., al 2021). FMG enables bringing the DRM out of this explanation circularity.

Finally, it is crucial to note that the potential applications of FMG are not restricted to words but can also extend to the non-verbal domain. Previous studies have already demonstrated the validity of investigating visual memory distortions with DRM (Děchtěrenko et al., 2021; Rodio et al., 2024). By feeding FMG with vector space trained on images or sounds, the system can easily arrange the sensory stimuli into lists optimized to evaluate the effect of auditory or visual similarity in triggering the DRM false memory effect. This flexibility of FMG enables researchers to potentially investigate false memory phenomena across a very large range of domains.

Looking forward, it is plausible to anticipate the emergence of new representation systems based on such multidimensional structures. FMG will be easily applicable to such advancements opening new research avenues that go well beyond the current possibilities.

## Data Availability

To make the FMG tool presented here easily accessible to as wide an audience as possible, we release an openly available tool to conveniently and intuitively work with it and a manual detailing the instructions of the FMG. Users can utilize the full range of functionalities by running the source code on MATLAB or by using the standalone application for Microsoft Windows without requiring any programming experience. The FMG software and the manual are available at https://osf.io/gsrfu.

## Footnotes

1 Note that here we used the term “Critical Lure” referring to all the new items falling within the close space defined by the Studied Items.

2 When only one critical lure is selected this option gives the possibility to have this item as the one closest to the centroid of the Studied Items compared to any other item, as in classical DRM lists. When choosing multiple Critical Lures, this setting ensures that only the first Critical Lure is the item closest to the centroid, while the remaining Critical Lures will still be within the close space but with variable distance to the centroid.

3 The choice to employ logarithmically spaced amplitudes is driven by the exponential increase in the number of items in a uniformly distributed space as the distance from a central point increases. Logarithmic scaling tends to correct for this effect, resulting in a more linear distribution of distances of New Items from the centroid of the Studied Items.

4 The database by Amenta et a., (in prep) includes nouns, adjectives and verbs taken from SUBTLEX-IT (http://crr.ugent.be/subtlex-it/), cleaned by excluding low frequent entries (i.e., Zipf values < 3; van Heuven et al., 2014), single letters, words whose lemma is tagged as unknown (i.e., foreign words, proper nouns, acronyms, and multiword utterances), plurals, and verbs in non-infinite form.

5 This value can be computed by applying the exponential function to the half of the ΔAIC between two models. In this case, specifically: exp(16 * .5).
